# Discrimination based on gender identity against transgender women and travestis in Brazil: a latent class analysis and associated factors

**DOI:** 10.1590/1980-549720240012.supl.1

**Published:** 2024-08-19

**Authors:** Laio Magno, Beo Oliveira Leite, Sandro Sperandei, Marcos Pereira, Daniela Riva Knauth, Andréa Fachel Leal, Maria Amélia de Sousa Mascena Veras, Inês Dourado, TransOdara Research Group

**Affiliations:** I Universidade do Estado da Bahia, Department of Life Sciences – Salvador (BA), Brazil.; II Universidade Federal da Bahia, Institute of Public Health – Salvador (BA), Brazil.; III Western Sydney University, Translational Health Research Institute – Penrith (NSW), Austrália.; IV Universidade Federal do Rio Grande do Sul, School of Medicine, Department of Social Medicine – Porto Alegre (RS), Brazil.; V Universidade Federal do Rio Grande do Sul, Institute of Philosophy and Human Sciences, Department of Sociology – Porto Alegre (RS), Brazil.; VI Santa Casa de São Paulo, School of Medical Sciences – São Paulo (SP), Brazil.

**Keywords:** Transgender persons, Transgender women, Discrimination, Gender identity, Latent class analysis, Brazil

## Abstract

**Objective:**

To identify groups of transgender women and *travestis* (TGW) with specific patterns of gender-based discrimination (GBD) and analyze the factors associated with GBD.

**Methods:**

A cross-sectional study was conducted with TGW recruited through respondent-driven sampling in five Brazilian cities (2019-2021). Latent class analysis was used to characterize GBD (low, medium, and high) using 14 observable variables. Descriptive analysis was performed, and associations between predictor variables and GBD were estimated by adjusted odds ratios (aOR) using ordinal logistic regression.

**Results:**

Out of a total of 1,317 TGW, 906 (68.8%) answered questions about GBD. Most were under 34 years old, single, and had a Brown race/skin color. GBD was classified as “low,” “medium,” and “high,” with estimates of 41.7, 44.5, and 13.8%, respectively. Variables positively associated with higher intensity of GBD included living in Manaus compared to São Paulo, being ≤34 years old compared to >34, being homeless compared to living in one’s own house or rented apartment, not having legally changed one’s name compared to those who had, and reporting physical or sexual violence compared to those who did not report. Variables negatively associated with higher intensity of GBD included having a Brown or Asian race/skin color compared to White and a monthly income ≥1 minimum wage compared to ³1.

**Conclusion:**

A high proportion of GBD was observed in Brazilian TGW, with this outcome associated with more vulnerable sociodemographic characteristics and a history of violence.

## INTRODUCTION

Transgender women and *travestis* (TGW) have been disproportionately affected by certain morbidities, notably HIV/AIDS and depression^1-3^. Specifically, when it comes to HIV/AIDS, a previous systematic review study revealed that discrimination is often associated with the three dimensions of vulnerability to HIV, *i.e*., individual (*e.g*., low condom use negotiation), social (*e.g*., obstacles to access schooling and formal employment), and programmatic (*e.g*., lack of access to information, prevention technologies, testing, and HIV counseling)^4^.

Several advancements have been made in recognizing the needs of TGW, such as the establishment of national LGBT health policies and the implementation of trans outpatient clinics and related services^5^, as well as the Federal Supreme Court’s interpretation equating crimes of LGBTphobia with those of racism^6^. However, studies show that this population often encounters numerous barriers to accessing healthcare services and receives inadequate support for their specific needs, especially concerning access to cross-hormone therapy, gender-affirming surgeries, and resistance from healthcare professionals regarding the use of their social names in the Brazilian Unified Health System (*Sistema Único de Saúde* – SUS)^7,8^.

From 2008 to 2021, Brazil was identified as the country with the highest number of recorded murders of transgender individuals worldwide^9^. Throughout this period, the country documented an annual mean of 123.8 murders, with a significant majority of trans youth victims aged 18 to 29 between 2017 and 2021. Furthermore, statistics reveal that 96% of these murders targeted TGW, while in 2021, 78% of victims identified as sex workers, with 81% being black and brown^10^.

Discrimination has been defined as a set of negative attitudes and behaviors against specific people or population groups, stemming from broader stigmatization processes^11-13^. It encompasses a set of institutionalized social expressions and relationships of domination and oppression whose intention is to maintain power and privileges within the system^14^. Gender-based discrimination (GBD) against trans people is derived from the existence of a hegemonic cis-heteronormative system, which perceives transgenders and *travestis* as “abnormal” or “immoral”, thereby imposing a set of social and economic disadvantages upon this population, as well as epidemiological challenges^4^.

GBD has been identified by numerous epidemiological and socio-anthropological studies as one of the main predictors of adverse health outcomes and barriers to accessing healthcare services^15-19^. Moreover, discrimination is documented even within the LGBT+ community itself^20^.

The literature highlights various approaches to analyzing data on GBD. A systematic review study showed that the variables commonly employed to determine the theoretical construct of “discrimination” are structured based on scales that have not been validated for the specific population of trans women^4^. Moreover, this study identified that the variables used in those reviewed studies were either derived from existing literature^21-25^, inspired by racial discrimination scales^26^, based on perceptions of discrimination related to the sexual orientation of men who have sex with men^27^, or influenced by homophobia^28,29^. Notably, some of these studies did not employ any statistical resources to develop their “discrimination” construct^23,24,30^.

It is known that certain methods commonly used to measure GBD have limitations, especially when the variables observed are dichotomous^31^ or not derived from validated scales. Thus, latent class analysis (LCA) emerges as a viable methodological approach. LCA identifies and categorizes latent (*i.e*., unobserved) classes of individuals with similar profiles based on responses provided in each of the dichotomous variables observed. By doing so, LCA seeks to identify groups of individuals who share similar response patterns across classification variables^31^, which is also referred to as *person-centered analysis*. This approach offers a plausible solution for synthesizing numerous dichotomous indicators, thereby facilitating the interpretation of theoretical constructs within quantitative data^32^.

Given the complexity of GBD, this study aimed to identify TGW subgroups with distinct patterns of discrimination based on gender identity and to analyze the associated factors.

## METHODS

### Study design, location, and population

TransOdara was a multicenter cross-sectional study conducted by a multidisciplinary team of researchers between December 2019 and July 2021. The study aimed to investigate the prevalence of sexually transmitted infections among transgender women and *travestis* in five Brazilian capitals: Campo Grande (MS – Central West region), Manaus (AM – North region), Porto Alegre (RS – South region), Salvador (BA – Northeast region), and São Paulo (SP – Southeast region).

Eligible TGW were those that met the following criteria:

1. Aged >18 years old;
2. Having been assigned male sex at birth and currently self-identifying with a female gender identity;
3. Being a resident of the metropolitan area of one of the study cities; and
4. Having a valid study participation coupon.

### Data collection and sampling

Participants were recruited using respondent-driven sampling (RDS)^33^ in each of the five cities. This recruitment was carried out through collaboration with social movements, activists, and health services dedicated to promoting human and LGBT rights in each city.

Before RDS recruitment began, qualitative formative research was carried out to understand the dynamics of the population in each location and to select “seeds”, that is, TGW who had social networks with a sufficient number of people to distribute coupons and who represented diversity pertaining a set of characteristics of interest to the study. Each “seed” received six coupons to invite other people from their social network, and these new invitees received an equal number of coupons. This process was repeated until the desired sample size was achieved in each city. The recruiter-recruited connection was monitored using a “coupon manager” system, allowing the entire recruitment chain to be traced through unique numeric identifiers. Participants in the study were provided two financial incentives: a primary one for reimbursement of food and transportation expenses to the data collection site; and a secondary reimbursement to encourage them to invite more participants to the study. The sample calculated for the study was 1,280 participants.

In the data collection venues in each city, participants were invited to fill out a structured questionnaire conducted by a trained interviewer. The questionnaire aimed to gather sociodemographic information, as well as details regarding gender affirmation procedures, sexual behavior, symptoms of sexually transmitted infections, and experiences of discrimination. All participants received counseling, educational materials, and condoms. More information can be found in Veras et al.^34^.

### Study variables

The outcome variable of the study was the latent variable DIG in the last 12 months (categorized as high, medium, and low). This variable was constructed using 14 observed variables related to self-reported experiences of discrimination in the 12 months prior to the interview. These variables were grouped into four dimensions (work, educational, private, and public), according to a theoretical review of the construct, and were similar to a previously published study^35^. Participants responded to questions with options including “often,” “sometimes,” “rarely,” “only once,” and “never,” which were grouped as “no” for those who answered “never” and “yes” for those who chose any of the other options:

a) Discrimination at work: was not selected or was fired from her job.
b) Educational discrimination: was mistreated or marginalized by teachers at school or college; was mistreated or marginalized by peers at school or college.
c) Private discrimination: was excluded or marginalized in a religious environment; was excluded or marginalized from a group of friends; was excluded or marginalized from a group of neighbors; was excluded or marginalized in her family environment.
d) Public discrimination: was blackmailed or subjected to extortion for money; received poor care in health services or by health professionals; was prevented from donating blood; was poorly attended to or mistreated in public services, such as hostels, sub-prefectures, transportation, or public bathrooms; was mistreated by police officers or poorly treated at police stations; was poorly served or denied entry to shops or recreational facilities; felt afraid of walking in public spaces.

Predictor variables were:

1. Sociodemographics — study site (Campo Grande, Manaus, Porto Alegre, Salvador, and São Paulo), age (35 years old or older, up to 34 years old), living situation (in their own or rented house or apartment, with friends, family or hotel, homeless, others), self-reported race/skin color (white, black, brown, Asian, and indigenous), education (elementary education, classified up to the 9th grade; secondary education, classified as complete high school, technical courses or incomplete higher education; complete higher education; or postgraduate degree), average monthly income (one or more minimum wages, less than one minimum wage), religion (no religion, Afro-Brazilian, evangelical, Catholic, spiritualist, and others), rectified name on official Brazilian document (yes, no);
2. Violence and incarceration in life — history of physical violence (no, yes), history of sexual violence (no, yes), history of incarceration in life (no, yes);
3. Behavioral — lifetime drug use (no, yes), steady partner in the last six months (no, yes), casual partner in the last six months (no, yes), business partner in the last six months (no, yes).

### Data analysis

Data from the five cities were consolidated into a unified database. The parameters of the LCA analysis — class prevalence and item response probabilities — were used to describe the latent classes of the final selected model. To determine the best model, the Bayesian information criterion (BIC) and the Akaike information criterion (AIC) were employed, allowing the comparison of models concerning the balance between fit and parsimony, with lower values indicating better fit. Furthermore, the selection of the model was influenced by the interpretability of the latent classes. Models ranging from two to six latent classes were compared using AIC and BIC. The proportions of the latent variable GBD and their respective 95% confidence intervals (CI) were estimated. A descriptive analysis of the population profile was carried out. Furthermore, the association between the predictor variables and the GBD outcome variable was analyzed, starting with bivariate analysis using an ordinal logistic regression model. For multivariate modeling, the process began with the complete model, encompassing all predictor variables, which were then removed one by one, with readjustment of the model, until the best fit was attained^36^. The final adjusted model was compared with the original full model using the maximum likelihood test. In both the multivariate and bivariate modeling stages, RDS weights were not considered, following the recommendation of Sperandei et al.^37^. All analyses were performed in R language, version 4.2.3^38^, with the MASS^39^ and poLCA^40^ packages.

### Ethical aspects

The project was approved by the Research Ethics Committee of the Santa Casa de Misericórdia de São Paulo (CAAE 05585518.7.0000.5479; opinion n°: 3.126.815 – 30/01/2019), as well as by other participating institutions. All participants included in the study signed the Informed Consent.

## RESULTS

Out of the total of 1,317 TGW recruited, 906 (68.8%) responded to the questions about GBD. Of these, the majority were from Manaus (33.8%), young individuals aged up to 34 years old (63.6%), single (71.7%), living in their own or rented house or apartment (59.9%), self-reported as mixed race/skin color (45.6%), with secondary education (70.3%), monthly income of one or more minimum wage (74.8%), without religion (35.2%), and who had not rectified their namo on official documents (72.4%). With regard to previous experiences of violence and interactions with health services, a significant majority reported having suffered sexual violence (51.0%), while the minority reported having experienced physical violence (14.2%), and having had difficulties in accessing healthcare services (10.4%). In terms of behavioral characteristics, the majority reported having used drugs in their lifetime (58.4%), while the minority reported having had a steady partner in the last six months (47.5%), a casual partner in the last six months (43.3%), and commercial partner in the last six months (36.5%) (Table 1).

**Table 1:** Characteristics of the population of transgender women and *travestis* in five Brazilian cities, 2019–2021.

Characteristics	Total (n=906)	Discrimination by gender identity		
		Low (n=378, 41.7%)	Medium (n=403, 44.5%)	High (n=125, 13.8%)
	n (%)	n (%)	n (%)	n (%)
Sociodemographic				
Study site				
São Paulo	211 (23.3)	109 (51.7)	86 (40.8)	16 (7.6)
Porto Alegre	78 (8.6)	32 (41.0)	39 (50.0)	7 (9.0)
Salvador	196 (21.6)	96 (49.0)	85 (43.4)	15 (7.7)
Manaus	306 (33.8)	87 (28.4)	148 (48.4)	71 (23.2)
Campo Grande	115 (12.7)	54 (47.0)	45 (39.1)	16 (13.9)
Age				
35 years old or older	330 (36.4)	172 (52.1)	119 (36.1)	39 (11.8)
Up to 34 years old	576 (63.6)	206 (35.8)	284 (49.3)	86 (14.9)
Marital status				
Single	650 (71.7)	265 (40.8)	287 (44.2)	98 (15.1)
In a relationship	132 (14.6)	52 (39.4)	59 (44.7)	21 (15.9)
Married/stable union	123 (13.6)	61 (49.6)	56 (45.5)	6 (4.9)
Did not respond	1 (0.1)	0 (0.0)	1 (100.0)	0 (0.0)
Housing situation				
In their own or rented house/apartment	543 (59.9)	252 (46.4)	233 (42.9)	58 (10.7)
With friends, family or in a hotel	274 (30.2)	104 (38.0)	124 (45.3)	46 (16.8)
Homeless	52 (5.7)	12 (23.1)	28 (53.8)	12 (23.1)
Others	37 (4.1)	10 (27.0)	18 (48.6)	9 (24.3)
Race/skin color				
White	203 (22.4)	79 (38.9)	91 (44.8)	33 (16.3)
Black	251 (27.7)	97 (38.6)	112 (44.6)	42 (16.7)
Brown	413 (45.6)	183 (44.3)	189 (45.8)	41 (9.9)
Asian	21 (2.3)	13 (61.9)	3 (14.3)	5 (23.8)
Indigenous	12 (1.3)	3 (25.0)	5 (41.7)	4 (33.3)
Did not respond	6 (0.7)	3 (50.0)	3 (50.0)	0 (0.0)
Education				
Elementary education	222 (24.5)	86 (38.7)	112 (50.5)	24 (10.8)
High school	637 (70.3)	271 (42.5)	275 (43.2)	91 (14.3)
Higher education or postgraduation	45 (5.0)	20 (44.4)	15 (33.3)	10 (22.2)
Did not respond	2 (0.2)	1 (50.0)	1 (50.0)	0 (0.0)
Average monthly income				
One or more minimum wages	678 (74.8)	274 (40.4)	318 (46.9)	86 (12.7)
Less than one minimum wage	118 (13.0)	67 (56.8)	38 (32.2)	13 (11.0)
Did not respond	110 (12.1)	37 (33.6)	47 (42.7)	26 (23.6)
Religion				
Without religion	319 (35.2)	136 (42.6)	145 (45.5)	38 (11.9)
Afro-Brazilian	192 (21.2)	74 (38.5)	95 (49.5)	23 (12.0)
Evangelical	73 (8.1)	34 (46.6)	31 (42.5)	8 (11.0)
Catholic	259 (28.6)	105 (40.5)	103 (39.8)	51 (19.7)
Spiritist	53 (5.8)	24 (45.3)	24 (45.3)	5 (9.4)
Others	7 (0.8)	3 (42.9)	4 (57.1)	0 (0.0)
Did not respond	3 (0.3)	2 (66.7)	1 (33.3)	0 (0.0)
Rectified name on official documents				
Yes	249 (27.5)	138 (55.4)	88 (35.3)	23 (9.2)
No	656 (72.4)	240 (36.6)	314 (47.9)	102 (15.5)
Did not respond	1 (0.1)	0 (0.0)	1 (100.0)	0 (0.0)
Violence and healthcare services				
Physical violence				
No	770 (85.0)	347 (45.1)	329 (42.7)	94 (12.2)
Yes	129 (14.2)	26 (20.2)	73 (56.6)	30 (23.3)
Did not respond	7 (0.8)	5 (71.4)	1 (14.3)	1 (14.3)
Sexual violence				
No	438 (48.3)	226 (51.6)	173 (39.5)	39 (8.9)
Yes	462 (51.0)	150 (32.5)	227 (49.1)	85 (18.4)
Did not respond	6 (0.7)	2 (33.3)	3 (50.0)	1 (16.7)
Problems accessing healthcare services				
No, but has not looked for these services	302 (33.3)	125 (41.4)	140 (46.4)	37 (12.3)
No, and looked for these services	503 (55.5)	232 (46.1)	213 (42.3)	58 (11.5)
Yes	94 (10.4)	18 (19.1)	47 (50.0)	29 (30.9)
Did not respond	7 (0.8)	3 (42.9)	3 (42.9)	1 (14.3)
Incarceration history				
No	704 (77.7)	307 (43.6)	305 (43.3)	92 (13.1)
Yes	198 (21.9)	70 (35.4)	98 (49.5)	30 (15.2)
Did not respond	4 (0.4)	1 (25.0)	0 (0.0)	3 (75.0)
Behavioral				
Lifetime use of drugs				
No	377 (41.6)	165 (43.8)	159 (42.2)	53 (14.1)
Yes	529 (58.4)	213 (40.3)	244 (46.1)	72 (13.6)
Paid sex				
No	253 (27.9)	108 (42.7)	108 (42.7)	37 (14.6)
Yes, only in the past	297 (32.8)	123 (41.4)	127 (42.8)	47 (15.8)
Yes, part time	170 (18.8)	66 (38.8)	80 (47.1)	24 (14.1)
Yes, full time	184 (20.3)	80 (43.5)	88 (47.8)	16 (8.7)
Did not respond	2 (0.2)	1 (50.0)	0 (0.0)	1 (50.0)
Permanent partner in the last six months				
No	473 (52.2)	196 (41.4)	203 (42.9)	74 (15.6)
Yes	430 (47.5)	182 (42.3)	197 (45.8)	51 (11.9)
Did not respond	3 (0.3)	0 (0.0)	3 (100.0)	0 (0.0)
Casual partner in the last six months				
No	510 (56.3)	205 (40.2)	228 (44.7)	77 (15.1)
Yes	392 (43.3)	170 (43.4)	174 (44.4)	48 (12.2)
Did not respond	4 (0.4)	3 (75.0)	1 (25.0)	0 (0.0)
Paying partner in the last six months				
No	572 (63.1)	239 (41.8)	244 (42.7)	89 (15.6)
Yes	331 (36.5)	137 (41.4)	158 (47.7)	36 (10.9)
Did not respond	3 (0.3)	2 (66.7)	1 (33.3)	0 (0.0)

In LCA, the three-class model was chosen. The classes were labeled as “low,” “medium,” and “high” GBD, based on the distribution of probabilities. Although the three-class model did not have better entropy (76%) and AIC (13,967.11) values, it did have better BIC (14,178.71) and adjusted BIC (14,338.57) values (Table 2).

**Table 2:** Latent class analysis diagnostic tests, 2–6 classes.

Diagnostic tests	2 classes	3 classes	4 classes	5 classes	6 classes
Akaike (AIC)	14,220.71	13,967.11	13,954.56	13,911.86	13,899.26
Bayesian (BIC)	14,360.17	14,178.71	14,238.29	14,267.73	14,327.27
BIC-sample-size adjusted	14,465.53	14,338.57	14,452.65	14,536.58	14,650.62
Entropy (%)	82	76	71	73	64

**Figure 1:**
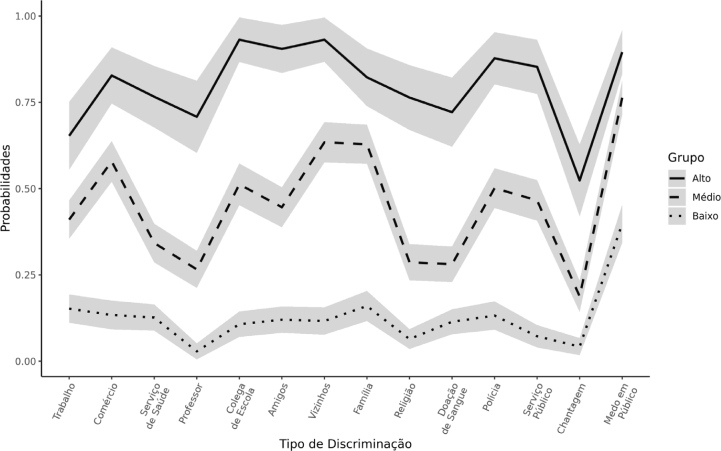
Gender identity discrimination model with three latent classes, according to the probability of inclusion in the classes based on the affirmative response to the item, in the population of transgender women and *travestis* in five Brazilian cities, 2019–2021.

The distribution of the observed GBD variables revealed significant instances of discrimination across various dimensions. Regarding work, 33.8% of TGW were either not selected for a job or were fired due to GBD-related reasons; in the educational sphere, 23.1% experienced mistreatment or marginalization by teachers at school or university, while 40.4% faced similas treatment from colleagues at school or university; regarding discrimination in private relationships, 26.3% were excluded or marginalized in religious environments, 37.6% were excluded from groups of friends or marginalized by them, 46.1% were excluded from groups of neighbors or marginalized by them, and 46.1% were excluded or marginalized in their family environment; in the public sphere, 17.5% reported instances of blackmail or extortion, 31.3% were poorly treated in healthcare services or by health professionals, 27.5% were prevented from donating blood, 35.8% were poorly treated or mistreated in public services, 40.2% were mistreated by police officers or poorly treated at police stations, 42.9% were poorly served or prevented from entering commercial establishments or leisure facilities, and 63.0% felt afraid of walking in public spaces (Figure 1 and Table 3).

The shaded area represents a standard error.

**Table 3:** Gender-based discrimination model with three latent classes according to the probability of inclusion in the classes based on the affirmative response to the item, in the population of transgender women and *travestis* in five Brazilian cities, 2019–2021.

Characteristics	Total			Low (n=378; 41.7%)			Medium (n=403; 44.5%)			High (n=125; 13.8%)		
	%	LL*	UL*	%	LL*	UL*	%	LL*	UL*	%	LL*	UL*
Discrimination at work												
Was not selected or was fired from her job	33.8	30.7	36.9	15.2	11.1	19.3	41.0	35.4	46.6	65.3	55.4	75.2
Educational discrimination												
Was mistreated or marginalized by teachers at school or college	23.1	20.3	25.8	2.9	0.5	5.2	26.6	21.2	32.0	70.8	60.3	81.3
Was mistreated or marginalized by peers at school or college	40.4	37.2	43.6	10.7	7.0	14.4	51.2	45.2	57.3	93.1	86.7	99.6
Private discrimination												
Was excluded or marginalized in a religious environment	26.3	23.4	29.1	6.4	3.5	9.3	28.7	23.4	33.9	76.4	67.0	85.8
Was excluded or marginalized from a group of friends	37.6	34.5	40.8	12.0	8.2	15.8	44.6	38.8	50.4	90.5	83.5	97.4
Was excluded or marginalized from a group of neighbors	46.1	42.9	49.4	11.7	7.7	15.6	63.4	57.6	69.3	93.1	86.7	99.6
Was excluded or marginalized in her family environment	46.1	42.9	49.4	16.0	11.6	20.4	62.9	57.2	68.5	82.2	73.9	90.6
Public discrimination												
Was blackmailed or extorted for money	17.5	15.1	20.0	4.2	1.7	6.7	18.8	14.3	23.3	52.4	41.9	62.8
Was poorly attended to in health services or by health professionals	31.3	28.3	34.4	12.7	8.9	16.5	34.2	28.6	39.9	76.6	67.7	85.5
Was prevented from donating blood	27.5	24.6	30.4	11.4	7.8	15.1	28.1	22.9	33.3	72.2	62.1	82.2
Was poorly attended to or mistreated in public services, such as hostels, sub-prefectures, transportation, or public bathrooms	35.8	32.6	38.9	7.2	4.0	10.5	46.6	40.7	52.5	85.3	77.4	93.1
Was mistreated by police officers or poorly treated at police stations	40.2	37.0	43.4	13.2	9.1	17.4	50.1	44.4	55.9	87.8	80.2	95.3
Was poorly served or prevented from entering shops or leisure facilities	42.9	39.7	46.2	13.4	9.2	17.6	57.9	52.0	63.8	82.8	74.6	90.9
Felt afraid to walk in public spaces	63.0	59.9	66.2	39.8	34.3	45.3	76.3	71.4	81.3	89.6	83.2	96.0

* LL: lower limit of the 95% CI; UL: upper limit of the 95% CI.

The probability of belonging to the latent GBD classes was 41.7% (n=378) for low, 44.5% (n=403) for medium, and 13.8% (n=125) for high. Among TGW classified as low GBD, the probability was equal to or less than 16.0% across all variables defining the latent classes, except for the variable “felt afraid of walking in public spaces” (39.8%); those classified with average DIG had a probability of 18.8 to 63.4%, except for the variable “felt afraid of walking in public spaces” (76.3%); and those classified as high DIG had a probability greater than 65%, except for the variable “was blackmailed or suffered extortion of money” (52.4%) (Table 3).

In the multivariate analysis, several variables exhibited a positive association with a higher intensity of GBD, namely: living in Manaus compared to living in São Paulo (OR=2.61; 95%CI 1.70–4.04); being less than or equal to 34 years old compared to being over 34 years old (OR=1.58; 95%CI 1.17–2.14); being homeless compared to those who live in their own or rented house or apartment (OR=2.28; 95%CI 1.17–4.44); not having updated their name on official documents compared to those who have (OR=1.62; 95%CI 1.15–2.30); and having reported physical (OR= 2.53; 95%CI 1.70–3.77) or sexual violence (OR=2.69; 95%CI 2.01–3.60) compared to those who did not report such experiences. On the other hand, the variables negatively associated with greater intensity of GBD were: having brown skin color (OR=0.64; 95%CI 0.44–0.93) or identifying as Asian or indigenous (OR=0.12; 95%CI 0 .03–0.42) compared to white; and average monthly income below one minimum wage compared to earning one or more salaries (OR=0.65; 95%CI 0.42–0.99) (Table 4).

**Table 4:** Factors associated with discrimination based on gender identity among transgender women and *travestis* in five Brazilian cities, 2019–2021.

Characteristic	Multivariate analysis*		
	OR	LL	UL
Sociodemographic			
Study site			
São Paulo	1.00		
Porto Alegre	1.24	0.70	2.17
Salvador	0.94	0.60	1.48
Manaus	2.61	1.70	4.04
Campo Grande	1.43	0.87	2.36
Age (years)			
35 or more	1.00		
Up to 34	1.58	1.17	2.14
Housing situation			
In their own or rented house/apartment	1.00		
With friends, family or in a hotel	0.97	0.69	1.36
Homeless	2.28	1.17	4.44
Others	2.01	0.92	4.37
Race/skin color			
White	1.00		
Black	1.03	0.68	1.57
Brown	0.64	0.44	0.93
Asian	0.12	0.03	0.42
Indigenous	1.31	0.36	4.69
Average monthly income			
One or more minimum wages	1.00		
Less than one minimum wage	0.65	0.42	0.99
Rectified name on official documents			
Yes	1.00		
No	1.62	1.15	2.30
Violence and healthcare services			
Physical violence			
No	1.00		
Yes	2.53	1.70	3.77
Sexual violence			
No	1.00		
Yes	2.69	2.01	3.60

* Ordinal logistic regression with comparisons of GBD categories: low *vs*. medium/high and low/medium *vs*. high. LL: lower limit of the 95% CI; UL: upper limit of the 95% CI.

## DISCUSSION

More than half of the TGW were classified into the medium and high GBD groups. The forms of GBD most likely to be responded to across classes were fear of walking in public spaces and experiences of exclusion or marginalization by neighbors or within the family environment. Moreover, the high latent class of GBD had response probabilities above 60% for nearly all items. Factors such as the municipality of residence, younger age, homelessness, lack of name rectification, and experiences of physical or sexual violence heightened the likelihood of high levels of discrimination. However, TGW who identified as mixed race or brown, or as Asian or indigenous, and with low income were less likely to experience high GBD.

Brazil, one of the countries with the highest rates of transgender deaths, particularly among TGW, serves as a backdrop for the various facets of transphobia entrenched within a cis-heteronormative system normalized within society^9,10^. Numerous other studies conducted with TGW in Brazil have highlighted incidents of GBD endured in spaces also identified in this study. The narratives of TGWs frequently encompass experiences of verbal, physical, or psychological violence, as well as exclusion and/or marginalization within their familial or communal contexts — often compelling them to leave their homes. Additionally, other spaces have also been reported as places of discrimination, such as in schools — resulting in school dropouts — or difficulties in accessing formal employment. Beyond these social spheres, TGW face heightened risks of violence and marginalization on the streets^41-43^, which may elucidate the associations between reports of physical or sexual violence and an increased likelihood of experiencing GBD, as documented in this study.

Similarly, this study revealed that homeless TGW were more likely to be categorized in the high GBD group. Increased exposure to cis-heteronormative environments, lack of social support, or heightened vulnerability to violence and harassment in public spaces could serve as plausible explanations for this phenomenon^15,41,44^. It is also worth mentioning that these same TGW were likely already vulnerable and suffering high GBD due to confinement and marginalization that compelled them to leave their familial, social, and community support networks, resulting in the loss of connections and ties^45,46^.

It should also be acknowledged that the sociocultural context may also play a role in the occurrence of GBD in this population. In this context, TGW residing in Manaus presented greater chances of experiencing GBD, possibly due to structural stigma, violence, conservatism, and entrenched sexist perspectives reinforcing male domination. These various forms of deep-rooted stigma and discrimination against the LGBT population have been documented in studies carried out in this city^47-49^. Future studies are needed to provide a more comprehensive understanding of GBD in TGW in Manaus.

Younger TGW (*i.e*., aged up to 34 years old) also had a greater chance of high GBD. Several factors may account for this association: possible internalization of GBD as a self-protection mechanism among older TGW, as repeated exposure to violence could render discriminatory events less conspicuous when compared to their younger counterparts^50^; conversely, younger TGW, who emerged from generations characterized by heightened political engagement and benefit from the longstanding advocacy efforts of the trans movement, may have greater awareness and a heightened perception regarding discriminatory acts^51^. A noteworthy example is the significant increase in the candidacy of trans individuals, particularly TGW, in Brazilian politics^52^.

The lack of social name recognition is also documented as one of the factors associated with GBD. From this perspective, studies carried out in Brazil^7,53,54^ and other countries^55^ already record the denial or non-recognition of the gender identity of TGW, a result of possible effects of structural, institutional, and interpersonal stigma. Failure to recognize the social name or female pronoun can have a negative impact, constraining access to services, schools, and formal employment opportunities^4,56^.

The findings from this study underscore the considerable impact of factors associated with the increase in GBD faced by TGW. An association between this outcome and more vulnerable sociodemographic characteristics and history of violence was observed. These findings not only illustrate the complexity of this issue but also point to the urgent need for targeted interventions addressing these disparities. Developing strategies that recognize and address the nuances of these experiences is crucial to promoting equity and mitigating the vulnerability faced by these communities. By understanding the intersection of factors that contribute to gender discrimination, more inclusive and effective policies can be informed.

This study had some limitations. Firstly, its cross-sectional design impedes the incorporation of temporal relationships into the associations studied. Additionally, the design of the RDS may include selection bias through non-probability sampling and network homophily, although it facilitates investigations into hard-to-reach populations. The indicators used to measure GBD rely on self-reported responses by TGW, which may underestimate real discrimination if it goes unnoticed or uninternalized. Moreover, other forms of discrimination (*e.g*. based on race/skin color, geographical region, generational differences, etc.) that could influence this perception were not measured. Furthermore, the ordinal logistic regression model employed does not account for classification errors of individuals into the classes of the latent variable, albeit it serves as a proxy for the factors associated with GBD.
