# Supplementary material for: Discrimination based on gender identity against transgender women and travestis in Brazil: a latent class analysis and associated factors
Source: Rev Bras Epidemiol. 2024 Aug 19;27(Suppl 1):e240012.supl.1. doi: 10.1590/1980-549720240012.supl.1 (PMC11338540; doi:10.1590/1980-549720240012.supl.1)
Supplement: Translation

# Translation: Discriminação por identidade de gênero entre mulheres trans e travestis no Brasil: uma análise de classes latentes e fatores associados

**DOI:** 10.1590/1980-549720240012.supl.1.2

**Authors:** Laio Magno, Beo Oliveira Leite, Sandro Sperandei, Marcos Pereira, Daniela Riva Knauth, Andréa Fachel Leal, Maria Amélia de Sousa Mascena Veras, Inês Dourado, TransOdara Research Group

**Affiliations:** I Universidade do Estado da Bahia, Departamento de Ciências da Vida – Salvador (BA), Brasil.; II Universidade Federal da Bahia, Instituto de Saúde Coletiva – Salvador (BA), Brasil.; III Western Sydney University, Translational Health Research Institute – Penrith (NSW), Austrália.; IV Universidade Federal do Rio Grande do Sul, Faculdade de Medicina, Departamento de Medicina Social – Porto Alegre (RS), Brasil.; V Universidade Federal do Rio Grande do Sul, Instituto de Filosofia e Ciências Humanas, Departamento de Sociologia – Porto Alegre (RS), Brasil.; VI Santa Casa de São Paulo, Faculdade de Ciências Médicas – São Paulo (SP), Brasil.

**Keywords:** Pessoas transgênero, Mulheres trans, Discriminação, Identidade de gênero, Análise de classes latentes, Brasil

## Abstract

**Objetivo:**

Identificar grupos de mulheres trans e travestis (MTT) com padrões específicos de discriminação por identidade de gênero (DIG) e analisar os fatores associados à DIG.

**Métodos:**

Estudo transversal com MTT recrutadas por *respondent-driven sampling* em cinco capitais brasileiras (2019-2021). Análise de classes latentes foi usada para caracterizar a DIG (em baixa, média e alta) usando 14 variáveis observáveis. Análise descritiva foi realizada e as associações entre variáveis preditoras e DIG foram estimadas por *odds ratio* ajustados (ORaj), usando regressão logística ordinal.

**Resultados:**

Do total de 1.317 MTT, 906 (68,8%) responderam perguntas sobre DIG. A maioria apresentava idade ≤34 anos, solteiras e com raça/cor de pele parda. DIG foi classificada em “baixa”, “média” e “alta”, com estimativas de 41,7, 44,5, 13,8%, respectivamente. As variáveis positivamente associadas à maior intensidade de DIG foram: morar em Manaus em comparação com São Paulo; possuir idade £34 anos em comparação com idade >34; estar em situação de rua em comparação com quem mora em casa ou apartamento próprio ou alugado; não ter retificado o nome em documento em comparação com quem retificou; e relato de violência física ou sexual em comparação com quem não relatou. As variáveis negativamente associadas à maior intensidade de DIG foram: raça/cor de pele parda ou amarela em comparação com branca; e renda média mensal <1 salário mínimo em comparação com ≥1 salários.

**Conclusão:**

Observaram-se alta proporção de DIG em MTT brasileiras e associação desse desfecho com características sociodemográficas mais vulneráveis e histórico de violência.

## INTRODUÇÃO

As mulheres trans e travestis (MTT) têm sido afetadas desproporcionalmente por algumas morbidades, especialmente HIV/aids e depressão^1-3^. Especificamente, quando se trata do HIV/aids, um estudo prévio de revisão sistemática revelou que a discriminação está comumente associada às três dimensões de vulnerabilidade ao HIV, i.e., individual (e.g, baixa negociação do preservativo), social (e.g., barreiras à escolarização e emprego formal) e programática (e.g., falta de acesso a informação, tecnologias de prevenção, testagem e aconselhamento de HIV)^4^.

Observam-se diversas conquistas e avanços no reconhecimento de necessidades das MTT, tais como a política nacional de saúde LGBT e a implementação de ambulatórios trans e outros serviços^5^, bem como a interpretação do Supremo Tribunal Federal da equiparação do crime de LGBTfobia como crime de racismo^6^. Entretanto estudos mostram que essa população comumente enfrenta diversas barreiras de acesso aos serviços de saúde e um acolhimento inadequado às suas necessidades específicas, especialmente no que diz respeito ao acesso à hormonioterapia cruzada, à cirurgias de modificação corporal e ao uso do nome social no Sistema Único de Saúde (SUS) por profissionais dos serviços^7,8^.

De 2008 a 2021, o Brasil foi apontado como o país onde mais se registraram assassinatos de pessoas trans no mundo^9^. Nesse período, o país registrou uma média anual de 123,8 assassinatos, e a perda majoritária de jovens trans com idade de 18 a 29 anos entre 2017 e 2021. Além disso, observou-se que 96% dos assassinatos foram de MTT e, em 2021, 78% delas se consideravam profissionais do sexo e 81% pretas e pardas^10^.

A discriminação tem sido definida como um conjunto de atitudes e comportamentos negativos contra pessoas ou grupos populacionais, que é derivada de processos mais amplos de estigmatização^11-13^. A discriminação também é referida como um conjunto de expressões e relacionamentos sociais institucionalizados de dominação e opressão cuja intenção é a manutenção do poder e do sistema de privilégios^14^. A discriminação por identidade de gênero (DIG) contra pessoas trans é derivada da existência de um sistema cis-heteronormativo hegemônico, que compreende as transgeneridades e travestilidades como “anormalidade” ou “imoralidade”, impondo a essa população um conjunto de desvantagens sociais, econômicas e epidemiológicas^4^.

A DIG é apontada por diversos estudos epidemiológicos e socioantropológicos como um dos principais preditores desses desfechos de morbidade e acesso aos serviços de saúde^15-19^. Além disso, a discriminação é registrada até mesmo dentro da própria comunidade LGBT+^20^.

A literatura indica diferentes maneiras de analisar dados sobre DIG. Estudo de revisão sistemática mostrou que geralmente as variáveis utilizadas para determinar o constructo teórico “discriminação” são estruturadas com base em escalas não validadas para população de mulheres trans^4^. Além disso, neste estudo, identificou-se que as variáveis utilizadas nos estudos revisados sistematicamente foram elaboradas com base em revisão da literatura^21-25^ ou inspiradas em escalas de discriminação racial^26^, percepção de discriminação baseada na orientação sexual de homens que fazem sexo com homens^27^, ou mesmo de homofobia^28,29^. Entre esses estudos, alguns não utilizaram nenhum recurso estatístico para elaboração do constructo “discriminação”^23,24,30^.

Sabe-se que alguns métodos comumente utilizados para mensurar a DIG possuem limitações, especialmente quando as variáveis observadas são dicotômicas^31^ ou não provenientes de escalas validadas. Assim, a análise de classes latentes (ACL) pode ser um método aplicável nesses cenários, pois identifica e classifica grupos subjacentes de indivíduos com perfis semelhantes em classes latentes (i.e., não observadas) com base nas respostas dadas em cada uma das variáveis dicotômicas observadas. Desse modo, a ACL busca identificar grupos de indivíduos que compartilham perfis de resposta semelhantes em variáveis de classificação ^31^, também podendo ser chamada de *análise centrada na pessoa*. Tal estratégia é uma solução plausível para sumarização de muitos indicadores dicotômicos de maneira a facilitar a interpretação da teoria em dados quantitativos^32^.

Diante da complexidade da DIG, este estudo tem como objetivo identificar subgrupos de MTT com distintos padrões de discriminação por identidade de gênero e analisar os fatores associados.

## MÉTODOS

### Desenho, local e população do estudo

O TransOdara foi um estudo transversal multicêntrico, conduzido por uma equipe multidisciplinar de pesquisadores, entre dezembro de 2019 e julho de 2021. O estudo enfocou a prevalência de infecções sexualmente transmissíveis entre mulheres trans e travestis em cinco capitais brasileiras: Campo Grande (MS – região Centro-Oeste), Manaus (AM – região Norte), Porto Alegre (RS – região Sul), Salvador (BA – região Nordeste) e São Paulo (SP – região Sudeste).

As MTT elegíveis foram aquelas que apresentaram os seguintes critérios:

1. Ter idade >18 anos;
2. Ter sido atribuído o sexo masculino ao nascer e a autoidentificação atual com uma identidade de gênero feminina;
3. Ser residente na área metropolitana de uma das cidades do estudo; e
4. Ter um cupom de participação no estudo válido.

### Coleta de dados e amostragem

As participantes foram recrutadas usando amostragem dirigida por respondentes (em inglês, *respondent-driven sampling –* RDS)^33^ em cada uma das cinco cidades. Esse recrutamento foi realizado por meio de articulação com movimentos sociais, ativistas e serviços de saúde e de promoção de direitos humanos e LGBT em cada cidade.

Antes do início do recrutamento do RDS, uma pesquisa formativa de natureza qualitativa foi realizada para conhecimento das dinâmicas da população em cada localidade, bem como para realização da escolha de “sementes” (i.e., aquelas MTT que tinham redes de contatos sociais com um número suficiente de pessoas para a distribuição dos cupons e que apresentavam diversidade em relação a um conjunto de caraterísticas de interesse do estudo). Cada uma das “sementes” recebeu seis cupons para convidar outras pessoas da sua rede social, e essas novas convidadas receberam mais um número igual de cupons. Esse processo se repetiu até que se atingiu o tamanho final da amostra desejada em cada cidade. O monitoramento da conexão recrutadora-recrutada foi realizado por meio de um “gerenciador de cupons”, de modo que, ao final, toda a cadeia de recrutamento fosse identificada através de um número. Esse estudo previu dois incentivos financeiros: um primário relacionado ao ressarcimento para alimentação e transporte da participante até o local de coleta de dados; e um ressarcimento secundário para incentivá-la a levar mais participantes ao estudo. A amostra calculada para o estudo foi de 1.280 participantes.

Nos espaços de coleta em cada cidade, as participantes foram convidadas a preencher um questionário estruturado conduzido por um entrevistador treinado para obter informações sociodemográficas, bem como de procedimentos de afirmação de gênero, comportamento sexual, sintomas de infecções sexualmente transmissíveis e discriminação. Todas as participantes receberam aconselhamento, materiais educativos e preservativos. Mais informações podem ser encontradas em Veras et al.^34^.

### Variáveis do estudo

A variável desfecho do estudo foi a variável latente DIG nos últimos 12 meses (alta, média e baixa). Para construção dessa variável, foram utilizadas 14 variáveis observadas referentes ao autorrelato de discriminação nos 12 meses prévios à entrevista, agrupadas em quatro dimensões (âmbito do trabalho, educacional, privado e público), segundo revisão teórica do construto e semelhante a estudo publicado previamente^35^. As opções de resposta às perguntas eram “muitas vezes”, “algumas vezes”, “poucas vezes”, “somente uma vez” e “nunca”, que foram agrupadas como “não” para aquelas que responderam “nunca” e “sim” para aquelas que apontaram qualquer das outras opções:

a) Discriminação no trabalho: não foi selecionada ou foi demitida do emprego.
b) Discriminação educacional: foi maltratada ou marginalizada por professores na escola ou faculdade; foi maltratada ou marginalizada por colegas na escola ou faculdade.
c) Discriminação privada: foi excluída ou marginalizada em ambiente religioso; foi excluída ou marginalizada de grupo de amigos; foi excluída ou marginalizada de grupo de vizinhos; foi excluída ou marginalizada em seu ambiente familiar.
d) Discriminação pública: foi chantageada ou sofreu extorsão de dinheiro; foi mal atendida em serviços de saúde ou por profissionais de saúde; foi impedida de doar sangue; foi mal atendida ou maltratada em serviços públicos, como albergues, subprefeituras, transporte ou banheiros públicos; foi maltratada por policiais ou mal atendida em delegacias; foi mal atendida ou impedida de entrar em comércio ou locais de lazer; sentiu medo de caminhar em espaços públicos.

As variáveis preditoras foram:

1. Sociodemográficas — sítio do estudo (Campo Grande, Manaus, Porto Alegre, Salvador e São Paulo), idade (35 anos ou mais, até 34 anos), situação de moradia (em casa ou apartamento próprio ou alugado, vive com amigos, família ou hotel, em situação de rua, outras), raça/cor da pele autorreferida (branca, preta, parda, asiática, indígena), escolaridade (ensino fundamental, classificado até o 9^o^ ano; ensino médio, classificado em segundo grau completo, cursos técnicos ou superior incompleto; superior completo; ou pós-graduação), renda média mensal (um ou mais salários mínimos, menos que um salário mínimo), religião (sem religião, afro-brasileira, evangélica, católica, espírita, outras), retificou o nome em documento oficial brasileiro (sim, não);
2. Violência e encarceramento na vida — histórico de violência física (não, sim), histórico de violência sexual (não, sim), história de encarceramento na vida (não, sim);
3. Comportamentais — uso de drogas na vida (não, sim), parceiro fixo nos últimos seis meses (não, sim), parceiro casual nos últimos seis meses (não, sim), parceiro comercial nos últimos seis meses (não, sim).

### Análise de dados

Os dados das cinco cidades foram organizados em um único banco de dados. Os parâmetros da análise de ACL — prevalências de classe e probabilidades de resposta ao item — foram utilizados para descrição das classes latentes do modelo final selecionado. Como critérios para seleção do melhor modelo, utilizaram-se o critério de informação bayesiano (BIC) e o critério de informação de Akaike (AIC), que permitem comparar os modelos em relação ao balanceamento entre ajuste e parcimônia, com menores valores indicando melhor ajuste. Além disso, a escolha do modelo também foi baseada na interpretabilidade das classes latentes. Foram comparados modelos de duas a seis classes latentes, utilizando-se os critérios AIC e BIC. As proporções da variável latente DIG, bem como os seus respectivos intervalos de 95% de confiança (IC) foram estimados. Foi realizada análise descritiva do perfil da população. Além disso, foi analisada associação entre as variáveis preditoras e a variável desfecho DIG iniciando-se pela análise bivariada, utilizando um modelo de regressão logística ordinal. Para a modelagem multivariada, partiu-se do modelo completo, incluindo todas as variáveis preditoras, que foram sendo retiradas uma a uma, com reajuste do modelo, até que o melhor ajuste fosse alcançado^36^. O modelo final ajustado foi comparado com o modelo completo original pelo teste da máxima verossimilhança. Em ambas as etapas de modelagem (multi ou bivariada), os pesos RDS não foram considerados, seguindo a recomendação de Sperandei et al.^37^. Todas as análises foram realizadas em linguagem R, versão 4.2.3^38^, com os pacotes MASS^39^ e poLCA^40^.

### Aspectos éticos

O projeto foi aprovado pelo Comitê de Ética em Pesquisa (CEP) da Santa Casa de Misericórdia de São Paulo (CAAE 05585518.7.0000.5479 - N° parecer: 3.126.815 - 30/01/2019), assim como pelas demais instituições participantes. Todas as participantes incluídas no estudo assinaram o Termo de Consentimento Livre e Esclarecido.

## RESULTADOS

Do total de 1.317 MTT recrutadas, 906 (68,8%) responderam às questões sobre DIG. Dessas, a maioria foi de Manaus (33,8%), jovens com idade até 34 anos (63,6%), solteiras (71,7%), morando em casa ou apartamento próprio ou alugado (59,9%), cor de pele/raça autorreferida parda (45,6%), com escolaridade média (70,3%), renda mensal de um ou mais salários mínimos (74,8%), sem religião (35,2%) e sem ter realizado retificação do nome no documento (72,4%). No que diz respeito à experiência prévia de violência e serviços de saúde, a maioria referiu ter sofrido violência sexual (51,0%), enquanto a minoria referiu ter sofrido violência física (14,2%) e ter tido problemas para acessar serviços de saúde (10,4%). Sobre as características comportamentais, a maioria relatou uso de drogas na vida (58,4%), enquanto a minoria relatou ter tido parceiro fixo nos últimos seis meses (47,5%), parceiro casual nos últimos seis meses (43,3%) e parceiro comercial nos últimos seis meses (36,5%) (Tabela 1).

**Tabela 1:** Características da população de mulheres trans e travestis em cinco cidades brasileiras, 2019–2021.

Variável	Total (n=906)	Discriminação por identidade de Gênero		
		Baixo (n=378, 41,7%)	Médio (n=403, 44,5%)	Alto (n=125, 13,8%)
	n (%)	n (%)	n (%)	n (%)
Sociodemográficas				
Sítio do estudo				
São Paulo	211 (23,3)	109 (51,7)	86 (40,8)	16 (7,6)
Porto Alegre	78 (8,6)	32 (41,0)	39 (50,0)	7 (9,0)
Salvador	196 (21,6)	96 (49,0)	85 (43,4)	15 (7,7)
Manaus	306 (33,8)	87 (28,4)	148 (48,4)	71 (23,2)
Campo Grande	115 (12,7)	54 (47,0)	45 (39,1)	16 (13,9)
Idade				
35 anos ou mais	330 (36,4)	172 (52,1)	119 (36,1)	39 (11,8)
Até 34 anos	576 (63,6)	206 (35,8)	284 (49,3)	86 (14,9)
Estado conjugal				
Solteira	650 (71,7)	265 (40,8)	287 (44,2)	98 (15,1)
Em um relacionamento	132 (14,6)	52 (39,4)	59 (44,7)	21 (15,9)
Casada/união estável	123 (13,6)	61 (49,6)	56 (45,5)	6 (4,9)
Não respondeu	1 (0,1)	0 (0,0)	1 (100,0)	0 (0,0)
Situação de moradia				
Em casa ou apartamento próprio ou alugado	543 (59,9)	252 (46,4)	233 (42,9)	58 (10,7)
Vive com amigos, família ou hotel	274 (30,2)	104 (38,0)	124 (45,3)	46 (16,8)
Em situação de rua	52 (5,7)	12 (23,1)	28 (53,8)	12 (23,1)
Outras	37 (4,1)	10 (27,0)	18 (48,6)	9 (24,3)
Raça/cor de pele				
Branca	203 (22,4)	79 (38,9)	91 (44,8)	33 (16,3)
Preta	251 (27,7)	97 (38,6)	112 (44,6)	42 (16,7)
Parda	413 (45,6)	183 (44,3)	189 (45,8)	41 (9,9)
Asiática	21 (2,3)	13 (61,9)	3 (14,3)	5 (23,8)
Indígena	12 (1,3)	3 (25,0)	5 (41,7)	4 (33,3)
Não respondeu	6 (0,7)	3 (50,0)	3 (50,0)	0 (0,0)
Escolaridade				
Educação fundamental	222 (24,5)	86 (38,7)	112 (50,5)	24 (10,8)
Ensino médio	637 (70,3)	271 (42,5)	275 (43,2)	91 (14,3)
Ensino superior ou pós-graduação	45 (5,0)	20 (44,4)	15 (33,3)	10 (22,2)
Não respondeu	2 (0,2)	1 (50,0)	1 (50,0)	0 (0,0)
Renda média mensal				
Um ou mais salários mínimos	678 (74,8)	274 (40,4)	318 (46,9)	86 (12,7)
Menos que um salário mínimo	118 (13,0)	67 (56,8)	38 (32,2)	13 (11,0)
Não respondeu	110 (12,1)	37 (33,6)	47 (42,7)	26 (23,6)
Religião				
Sem religião	319 (35,2)	136 (42,6)	145 (45,5)	38 (11,9)
Afro-brasileira	192 (21,2)	74 (38,5)	95 (49,5)	23 (12,0)
Evangélica	73 (8,1)	34 (46,6)	31 (42,5)	8 (11,0)
Católica	259 (28,6)	105 (40,5)	103 (39,8)	51 (19,7)
Espírita	53 (5,8)	24 (45,3)	24 (45,3)	5 (9,4)
Outras	7 (0,8)	3 (42,9)	4 (57,1)	0 (0,0)
Não respondeu	3 (0,3)	2 (66,7)	1 (33,3)	0 (0,0)
Retificou o nome em documento				
Sim	249 (27,5)	138 (55,4)	88 (35,3)	23 (9,2)
Não	656 (72,4)	240 (36,6)	314 (47,9)	102 (15,5)
Não respondeu	1 (0,1)	0 (0,0)	1 (100,0)	0 (0,0)
Violência e serviços de saúde				
Violência física				
Não	770 (85,0)	347 (45,1)	329 (42,7)	94 (12,2)
Sim	129 (14,2)	26 (20,2)	73 (56,6)	30 (23,3)
Não respondeu	7 (0,8)	5 (71,4)	1 (14,3)	1 (14,3)
Violência sexual				
Não	438 (48,3)	226 (51,6)	173 (39,5)	39 (8,9)
Sim	462 (51,0)	150 (32,5)	227 (49,1)	85 (18,4)
Não respondeu	6 (0,7)	2 (33,3)	3 (50,0)	1 (16,7)
Problemas para acessar serviços de saúde				
Não, mas não procurei esses serviços	302 (33,3)	125 (41,4)	140 (46,4)	37 (12,3)
Não, e procurei esses serviços	503 (55,5)	232 (46,1)	213 (42,3)	58 (11,5)
Sim	94 (10,4)	18 (19,1)	47 (50,0)	29 (30,9)
Não respondeu	7 (0,8)	3 (42,9)	3 (42,9)	1 (14,3)
História de encarceramento na vida				
Não	704 (77,7)	307 (43,6)	305 (43,3)	92 (13,1)
Sim	198 (21,9)	70 (35,4)	98 (49,5)	30 (15,2)
Não respondeu	4 (0,4)	1 (25,0)	0 (0,0)	3 (75,0)
Comportamentais				
Uso de drogas na vida				
Não	377 (41,6)	165 (43,8)	159 (42,2)	53 (14,1)
Sim	529 (58,4)	213 (40,3)	244 (46,1)	72 (13,6)
Sexo comercial				
Não	253 (27,9)	108 (42,7)	108 (42,7)	37 (14,6)
Sim, apenas no passado	297 (32,8)	123 (41,4)	127 (42,8)	47 (15,8)
Sim, parcialmente	170 (18,8)	66 (38,8)	80 (47,1)	24 (14,1)
Sim, em tempo integral	184 (20,3)	80 (43,5)	88 (47,8)	16 (8,7)
Não respondeu	2 (0,2)	1 (50,0)	0 (0,0)	1 (50,0)
Parceiro fixo nos últimos seis meses				
Não	473 (52,2)	196 (41,4)	203 (42,9)	74 (15,6)
Sim	430 (47,5)	182 (42,3)	197 (45,8)	51 (11,9)
Não respondeu	3 (0,3)	0 (0,0)	3 (100,0)	0 (0,0)
Parceiro casual nos últimos seis meses				
Não	510 (56,3)	205 (40,2)	228 (44,7)	77 (15,1)
Sim	392 (43,3)	170 (43,4)	174 (44,4)	48 (12,2)
Não respondeu	4 (0,4)	3 (75,0)	1 (25,0)	0 (0,0)
Parceiro comercial nos últimos seis meses				
Não	572 (63,1)	239 (41,8)	244 (42,7)	89 (15,6)
Sim	331 (36,5)	137 (41,4)	158 (47,7)	36 (10,9)
Não respondeu	3 (0,3)	2 (66,7)	1 (33,3)	0 (0,0)

Na ACL, o modelo com três classes foi escolhido. As classes foram denominadas “baixa”, “média” e “alta” DIG, sendo designadas com base na distribuição das probabilidades. Embora o modelo com três classes não tenha tido melhores valores de entropia (76%) e AIC (13967,11), teve melhores valores de BIC (14178,71) e BIC ajustado (14338,57) (Tabela 2).

**Tabela 2:** Testes diagnósticos da análise de classes latentes, 2–6 classes.

Testes diagnósticos	2 classes	3 classes	4 classes	5 classes	6 classes
Akaike (AIC)	14220,71	13967,11	13954,56	13911,86	13899,26
Bayesiano (BIC)	14360,17	14178,71	14238,29	14267,73	14327,27
BIC-sample-size adjusted	14465,53	14338,57	14452,65	14536,58	14650,62
Entropia (%)	82	76	71	73	64

A distribuição das variáveis observadas de DIG mostrou que, na dimensão da discriminação no mundo do trabalho, 33,8% das MTT não foram selecionadas para um emprego ou foram demitidas por conta da DIG; na dimensão discriminação no âmbito educacional, 23,1% foram maltratadas ou marginalizadas por professores na escola ou na universidade e 40,4% foram maltratadas ou marginalizadas por colegas na escola ou na universidade; na dimensão discriminação no âmbito das relações privadas, 26,3% foram excluídas ou marginalizadas no ambiente religioso, 37,6% foram excluídas de grupos de amigos ou marginalizadas por eles, 46,1% foram excluídas de grupos de vizinhos ou marginalizadas por eles e 46,1% foram excluídas ou marginalizadas no seu ambiente familiar; na dimensão discriminação pública, 17,5% foram chantageadas ou sofreram extorsão de dinheiro, 31,3% foram mal atendidas em serviços de saúde ou por profissionais de saúde, 27,5% foram impedidas de doar sangue, 35,8% foram mal atendidos ou maltratadas em serviços públicos, 40,2% foram maltratadas por policiais ou mal atendidas em delegacias, 42,9% foram mal atendidas ou impedidas de entrar em estabelecimentos comerciais ou locais de lazer e 63,0% sentiram medo de caminhar em espaços públicos (Figura 1 e Tabela 3).

**Figura 1:** Modelo de discriminação por identidade de gênero com três classes latentes, de acordo com a probabilidade de inclusão nas classes com base na resposta afirmativa ao item, na população de mulheres trans e travestis em cinco cidades brasileiras, 2019–2021.

A área sombreada representa um erro-padrão.

**Tabela 3:** Modelo de discriminação por identidade de gênero com três classes latentes de acordo com a probabilidade de inclusão nas classes com base na resposta afirmativa ao item, na população de mulheres trans e travestis em cinco cidades brasileiras, 2019–2021.

Variáveis	Total			Baixa (n=378; 41,7%)			Média (n=403; 44,5%)			Alta (n=125; 13,8%)		
	%	LI*	LS*	%	LI*	LS*	%	LI*	LS*	%	LI*	LS*
Discriminação no trabalho												
Não foi selecionada ou foi demitida do emprego	33,8	30,7	36,9	15,2	11,1	19,3	41,0	35,4	46,6	65,3	55,4	75,2
Discriminação educacional												
Foi maltratada ou marginalizada por professores na escola ou faculdade	23,1	20,3	25,8	2,9	0,5	5,2	26,6	21,2	32,0	70,8	60,3	81,3
Foi maltratada ou marginalizada por colegas na escola ou faculdade	40,4	37,2	43,6	10,7	7,0	14,4	51,2	45,2	57,3	93,1	86,7	99,6
Discriminação privada												
Foi excluída ou marginalizada em ambiente religioso	26,3	23,4	29,1	6,4	3,5	9,3	28,7	23,4	33,9	76,4	67,0	85,8
Foi excluída ou marginalizada de grupo de amigos	37,6	34,5	40,8	12,0	8,2	15,8	44,6	38,8	50,4	90,5	83,5	97,4
Foi excluída ou marginalizada de grupo de vizinhos	46,1	42,9	49,4	11,7	7,7	15,6	63,4	57,6	69,3	93,1	86,7	99,6
Foi excluída ou marginalizada em seu ambiente familiar	46,1	42,9	49,4	16,0	11,6	20,4	62,9	57,2	68,5	82,2	73,9	90,6
Discriminação pública												
Foi chantageada ou sofreu extorsão de dinheiro	17,5	15,1	20,0	4,2	1,7	6,7	18,8	14,3	23,3	52,4	41,9	62,8
Foi mal atendida em serviços de saúde ou por profissionais de saúde	31,3	28,3	34,4	12,7	8,9	16,5	34,2	28,6	39,9	76,6	67,7	85,5
Foi impedida de doar sangue	27,5	24,6	30,4	11,4	7,8	15,1	28,1	22,9	33,3	72,2	62,1	82,2
Foi mal atendida ou mal tratada em serviços públicos, como albergues, subprefeituras, transporte ou banheiros públicos	35,8	32,6	38,9	7,2	4,0	10,5	46,6	40,7	52,5	85,3	77,4	93,1
Foi maltratada por policiais ou mal atendida em delegacias	40,2	37,0	43,4	13,2	9,1	17,4	50,1	44,4	55,9	87,8	80,2	95,3
Foi mal atendida ou impedida de entrar em comércio ou locais de lazer	42,9	39,7	46,2	13,4	9,2	17,6	57,9	52,0	63,8	82,8	74,6	90,9
Sentiu medo de caminhar em espaços públicos	63,0	59,9	66,2	39,8	34,3	45,3	76,3	71,4	81,3	89,6	83,2	96,0

*LI: limite inferior do IC95%; LS: limite superior do IC95%.

A probabilidade de pertencer às classes latentes de DIG foi de 41,7% (n=378) para baixa, 44,5% (n=403) para média e 13,8% (n=125) para alta. As MTT classificadas com baixa DIG tiveram uma probabilidade igual ou menor que 16,0% em todas as variáveis que compuseram as classes latentes, exceto para a variável “sentiu medo de caminhar em espaços públicos” (39,8%); aquelas classificadas com média DIG tiveram uma probabilidade de 18,8% a 63,4%, exceto para a variável “sentiu medo de caminhar em espaços públicos” (76,3%); e aquelas classificadas com alta DIG tiveram uma probabilidade maior que 65%, exceto para a variável “foi chantageada ou sofreu extorsão de dinheiro”(52,4%) (Tabela 3).

Na análise multivariada, as variáveis positivamente associadas à maior intensidade de DIG foram: morar em Manaus em comparação com morar em São Paulo (OR=2,61; IC95% 1,70–4,04); possuir idade menor ou igual a 34 anos em comparação com idade maior a 34 (OR=1,58; IC95% 1,17–2,14); estar em situação de rua em comparação com quem mora em casa ou apartamento próprio ou alugado (OR=2,28; IC95% 1,17–4,44); não ter retificado o nome em documento em comparação com quem retificou (OR=1,62; IC95% 1,15–2,30); e ter relatado de violência física (OR= 2,53; IC95% 1,70–3,77) ou sexual (OR=2,69; IC95% 2,01–3,60) em comparação com quem não relatou. Por outro lado, as variáveis negativamente associadas à maior intensidade de DIG foram: raça/cor de pele parda (OR=0,64; IC95% 0,44–0,93) ou amarela (OR=0,12; IC95% 0,03–0,42) em comparação com a branca; e renda média mensal menor que um salário mínimo em comparação com um ou mais salários (OR=0,65; IC95% 0,42–0,99) (Tabela 4).

**Tabela 4:** Fatores associados à discriminação baseada na identidade de gênero entre mulheres trans e travestis em cinco cidades brasileiras, 2019–2021.

Variável	Análise multivariada*		
	OR	LI	LS
Sociodemográficas			
Sítio do estudo			
São Paulo	1,00		
Porto Alegre	1,24	0,70	2,17
Salvador	0,94	0,60	1,48
Manaus	2,61	1,70	4,04
Campo Grande	1,43	0,87	2,36
Idade (anos)			
35 ou mais	1,00		
Até 34	1,58	1,17	2,14
Situação de moradia			
Em casa ou apartamento próprio ou alugado	1,00		
Vive com amigos, família ou hotel	0,97	0,69	1,36
Em situação de rua	2,28	1,17	4,44
Outras	2,01	0,92	4,37
Raça/cor de pele			
Branca	1,00		
Preta	1,03	0,68	1,57
Parda	0,64	0,44	0,93
Amarela	0,12	0,03	0,42
Indígena	1,31	0,36	4,69
Renda média mensal			
Um ou mais salários mínimos	1,00		
Menos que um salário mínimo	0,65	0,42	0,99
Retificou o nome em documento			
Sim	1,00		
Não	1,62	1,15	2,30
Violência e serviços de saúde			
Violência física			
Não	1,00		
Sim	2,53	1,70	3,77
Violência sexual			
Não	1,00		
Sim	2,69	2,01	3,60

*Regressão logística ordinal com as comparações das categorias da DIG: baixa vs. média/alta e baixa/média vs. alta. LI: limite inferior do IC95%; LS: limite superior do IC95%.

## DISCUSSÃO

Mais da metade das MTT foram classificadas nos grupos de média e alta DIG. As formas de DIG com maior probabilidade de resposta entre as classes foram o medo de andar em espaços públicos, ter sido excluída ou marginalizada por grupos de vizinhos ou no ambiente familiar. Além do mais, a classe latente alta DIG teve probabilidades de resposta acima de 60% para quase todos os itens. Fatores como o município de residência, menor idade, viver em situação de rua, não ter retificado e ter sofrido violência física ou sexual aumentaram a chance de alta discriminação. Entretanto as MTT que se autodeclararam pardas ou amarelas e com baixa renda tiveram menor chance de alta DIG.

O Brasil, um dos países com o maior registro de óbitos de pessoas trans, especialmente entre MTT, é cenário das diversas nuances da transfobia estruturada e admitida através de um sistema cis-heteronormativo naturalizado na sociedade^9,10^. Diversos outros estudos com MTT no Brasil têm demonstrado relatos de DIG sofridos em espaços também relatados neste estudo. Os discursos mais frequentes das MTT se relacionam com violência verbal, física ou psicológica, exclusão e(ou) marginalização dos seus ambientes familiar ou comunitário — o que muitas vezes as forçam a sair de casa. Outros espaços também têm sido relatados com presença de discriminação, como nas escolas — resultando no abando escolar — ou na dificuldade de acesso ao trabalho formal. Longe desses diversos espaços de sociabilidade, nas ruas, as MTT sofrem maior risco de violência e marginalização^41-43^, o que pode explicar associações entre relatos de sofrimento de violência física ou sexual e maior chance de sofrer DIG, conforme registadas neste estudo.

Da mesma forma, este estudo identificou que as MTT que viviam em situação de rua tinham maior chance de serem classificadas no grupo alta DIG. A maior exposição ao espaço cis-heteronormativo, a falta de suporte social ou o maior risco de violência e assédio nos espaços públicos podem ser possíveis explicações para este efeito^15,41,44^. Vale ressaltar também que é provável que essas mesmas MTT já estivessem vulnerabilizadas e sofrendo alta DIG devido à reclusão e marginalização que as forçaram a sair de seus núcleos familiares, de suporte social e comunitário e perda de vínculos^45,46^.

Destaque-se, ainda, que o contexto sociocultural também pode ser um fator associado a ocorrência de DIG nessa população. Nesse contexto, as MTT residentes em Manaus apresentaram maiores chances de DIG, possivelmente, devido a estigma estrutural, violência, conservadorismo e perspectivas machistas de dominação masculina em suas diferentes formas de estigma e discriminação arraigados contra a população LGBT, conforme documentada em estudos realizados nessa cidade^47-49^. Futuros estudos são necessários para fornecer uma compreensão mais abrangente da DIG em MTT em Manaus.

As MTT mais novas (i.e., com idade até 34 anos) também possuíram maior chance de alta DIG. Alguns fatores podem explicar tal associação: a possível internalização da DIG como mecanismo de autoproteção criado entre as MTT mais velhas, dada as repetidas exposições de violência, podem tornar eventos discriminatórios menos perceptíveis quando comparada com as MTT mais novas^50^; de uma outra forma, MTT mais jovens, que surgiram de gerações com maior engajamento político, assim como uma luta do movimento trans há anos articulado e direitos conquistados, podem influenciar maior consciência e percepção sobre atos discriminatórios^51^. Como exemplo, pode-se apontar o aumento expressivo de candidatura de pessoas trans, especialmente MTT para a política brasileira^52^.

A falta de reconhecimento do nome social também é documentada como um dos fatores associados à DIG. Nessa perspectiva, estudos no Brasil^7,53,54^ e em outros países^55^ já registram a negação ou o não reconhecimento da identidade de gênero das MTT, resultado de possíveis efeitos do estigma estrutural, institucional e interpessoal. O não reconhecimento do nome social ou do pronome feminino pode impactar negativamente, limitando o acesso a serviços, escolas, trabalho formal^4,56^.

Os resultados obtidos neste estudo destacam a significativa influência dos fatores associados ao aumento da DIG enfrentada pelas MTT. Observou-se uma associação desse desfecho com características sociodemográficas mais vulneráveis e histórico de violência. Esses achados não apenas ilustram a complexidade dessa questão, mas também apontam para a necessidade urgente de ações que sejam específicas a essas desproporcionalidades. Desenvolver estratégias que reconheçam e abordem as nuances dessas experiências é crucial para promover a equidade e mitigar a vulnerabilidade que essas comunidades enfrentam. Ao compreender-se a interseção de fatores que contribuem para a discriminação de gênero, pode-se informar políticas mais inclusivas e eficazes.

Este estudo possuiu algumas limitações. O estudo transversal dificulta a incorporação de relações temporais nas associações estudadas. O desenho do RDS pode incluir viés de seleção através da amostragem não probabilística e homofilia da rede, apesar de não impedir que tais investigações para populações de difícil acesso sejam realizadas. Os indicadores utilizados para mensurar a DIG representam respostas autorrelatadas pelas MTT e, portanto, podem subestimar discriminação real caso não tenha sido uma experiência percebida ou internalizada. Da mesma forma, outras experiências de discriminação (ex.: raça/cor da pele, região, geração etc.) que podem influenciar nessa percepção não foram aferidas. Além do mais, o modelo de regressão logística ordinal não incorpora os erros de classificação de indivíduos nas classes da variável latente, entretanto não impede que o modelo represente um *proxy* dos fatores associados com a DIG.
